# Optical and Electrical Performance of MOS-Structure Silicon Solar Cells with Antireflective Transparent ITO and Plasmonic Indium Nanoparticles under Applied Bias Voltage

**DOI:** 10.3390/ma9080682

**Published:** 2016-08-10

**Authors:** Wen-Jeng Ho, Ruei-Siang Sue, Jian-Cheng Lin, Hong-Jang Syu, Ching-Fuh Lin

**Affiliations:** 1 Department of Electro-Optical Engineering, National Taipei University of Technology, No. 1, Section 3, Zhongxial East Road, Taipei 10608, Taiwan; t103658020@ntut.edu.tw (R.-S.S.); t104658023@ntut.edu.tw (J.-C.L.); 2 Graduate Institute of Photonics and Optoelectronics, National Taiwan University, No. 1, Section 4, Roosevelt Road, Taipei 10617, Taiwan; f98941054@ntu.edu.tw (H.-J.S.); lincf@ntu.edu.tw (C.-F.L.)

**Keywords:** ITO-electrode, indium nanoparticles (In-NPs), plasmonics, MOS-structure solar cell

## Abstract

This paper reports impressive improvements in the optical and electrical performance of metal-oxide-semiconductor (MOS)-structure silicon solar cells through the incorporation of plasmonic indium nanoparticles (In-NPs) and an indium-tin-oxide (ITO) electrode with periodic holes (perforations) under applied bias voltage. Samples were prepared using a plain ITO electrode or perforated ITO electrode with and without In-NPs. The samples were characterized according to optical reflectance, dark current voltage, induced capacitance voltage, external quantum efficiency, and photovoltaic current voltage. Our results indicate that induced capacitance voltage and photovoltaic current voltage both depend on bias voltage, regardless of the type of ITO electrode. Under a bias voltage of 4.0 V, MOS cells with perforated ITO and plain ITO, respectively, presented conversion efficiencies of 17.53% and 15.80%. Under a bias voltage of 4.0 V, the inclusion of In-NPs increased the efficiency of cells with perforated ITO and plain ITO to 17.80% and 16.87%, respectively.

## 1. Introduction

Photovoltaic technology is helping to provide clean renewable energy. Most current photovoltaic devices are based on wafers of crystalline silicon (c-Si), and although the cost of bulk Si has steadily decreased over the past 10 years, this trend cannot continue indefinitely. This has prompted researchers to seek improvements in the efficiency of photovoltaic devices through the application of novel devices, such as hetero-structures [1,2,3], plasmonic light scattering [4,5,6,7,8], and cascade multi-junctions [9,10,11]. This has led to the development of nanostructural plasmonic silicon solar cells [12], nanostructural thin-film silicon solar cells using gold nanoparticles [13], and plasmonic effects of Au/Ag bimetallic multi-spiked nanoparticles [14]. Efficiency can also be increased using inversion-layer metal-insulator-semiconductors (IL-MIS) and metal-oxide-semiconductors (MOS) [15,16,17,18,19]. Researchers have reported that the photovoltaic performance of MIS or MOS solar cells (SC) can be enhanced through the application of bias voltage [20,21,22]; however, few studies have examined the effects of plasmonic metallic nanoparticles in conjunction with bias voltage on MOS solar cells.

In this study, we sought to improve the optical and electrical performance of Si solar cells with a MOS structure by taking advantage of the anti-reflective properties of indium-tin-oxide (ITO) in the fabrication of electrodes. We also investigated the effects of applying bias voltage to ITO electrodes and coating them with indium nanoparticles (In-NPs) to achieve plasmonic light scattering. Finally, we combined bias voltage with plasmonic effects and investigated the effects on photovoltaic performance. The performance of the resulting MOS-structure solar cells was evaluated according to the dark current voltage (I-V), optical reference, external quantum efficiency (EQE), and photovoltaic current density voltage (J-V).

## 2. Results and Discussion

**Figure 1:**
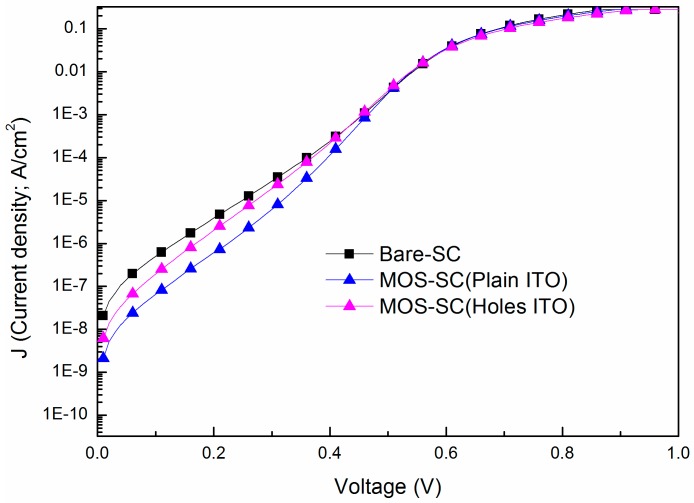
Dark I-V characteristics of bare cell and cells with MOS structure (using plain and perforated electrodes) at 25 °C.

Figure 1 presents the dark I-V characteristics of a bare cell and a cell with a MOS structure at 25 °C. The ideality factor (*n*) and reverse saturation current density (*J*_0_) extracted from the measured I-V curves are listed in Table 1. The *n* and *J*_0_ values of the bare cell were 1.87 and 6.14 × 10^−8^ A/cm^2^, respectively. The *n* and *J*_0_ values of the MOS cell with plain ITO (with periodic holes–type ITO) were 1.51 (1.65) and 3.84 × 10^−9^ (1.82 × 10^−8^) A/cm^2^, respectively. These improvements can be attributed to the suppression of surface recombination through the application of a TiO_2_ dielectric film on the surface of the cell.

**Figure 2:**
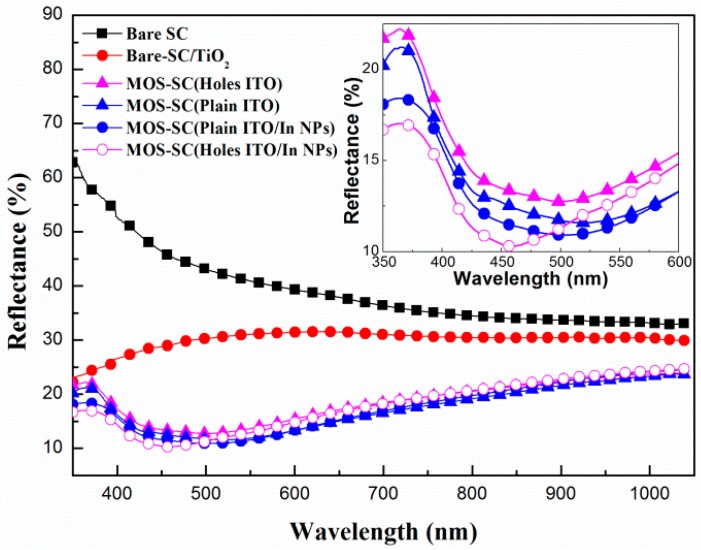
Optical reflectance of bare cell, cell with TiO_2_ film, MOS cell with plain ITO electrode, MOS cell with perforated ITO electrode, MOS cell with plain ITO electrode and In-NPs, and MOS cell with perforated ITO electrode and In-NPs.

**Figure 3:**
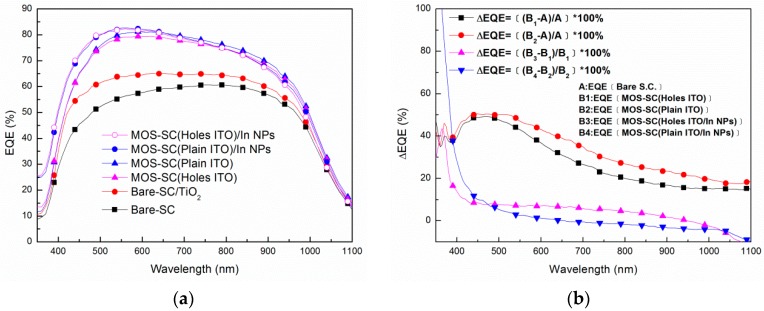
(**a**) EQE response of all solar cells tested in this study; (**b**) EQE enhancement can be attributed to anti-reflective properties and plasmonic light scattering.

Figure 2 presents the optical reflectance of the bare cell, the cell with a film of TiO_2_, the MOS cell with a plain ITO electrode, the MOS cell with a perforated ITO electrode, the MOS cell with In-NPs and a plain ITO electrode, and the MOS cell with In-NPs and a perforated ITO electrode. The reflectance values of cells with a TiO_2_ coating or ITO/TiO_2_ structure were lower than those of the bare cell, due to the anti-reflective properties of TiO_2_ and ITO/TiO_2_. The reflectance of the cell with a plain ITO electrode was lower than that of the cell with a perforated ITO electrode, due to the fact that the perforations reduced the total area of the ITO, thereby reducing the anti-reflective effects. The MOS cell with In-NPs presented a blue shift in the optical reflectance curves and a lower overall reflectance due to the plasmonic light scattering of the In-NPs. As shown in the inset of Figure 3, the incorporation of perforated electrodes blue-shifted the reflectance curves from 500 to 450 nm and reduced reflectance by 11% (at a wavelength of 450 nm).

Figure 3a presents the EQE response of the six solar cells tested in this study. The EQE values of the cells with a TiO_2_ layer and the cell with an ITO/TiO_2_ structure were higher than that of the bare cell, due to the anti-reflective properties of TiO_2_ and ITO/TiO_2_. The EQE of the cell with a plain ITO electrode was higher than that of the cell with a perforated ITO electrode, due to the fact that the perforations reduced the total area of the ITO, thereby reducing the anti-reflective effects. The MOS cell with In-NPs presented an impressive improvement in EQE at wavelengths of 350–600 nm. Overall, the EQE response values are in good agreement with the results of optical reflectance. Figure 3b illustrates the improvements in EQE under the effects of the anti-reflective coatings in conjunction with plasmonic light scattering in the In-NPs. The plain ITO electrode was more effective than the perforated ITO electrode in improving EQE enhancement across the entire range of wavelengths. We observed a peak at approximately 500 nm corresponding to a trough in the reflective spectrum, which is indicative of an improvement in EQE. At wavelengths below 500 nm, the In-NPs improved EQE values in all samples (regardless of the ITO electrode used); however, the cells with a perforated electrode presented a more pronounced improvement in EQE at wavelengths between 350 and 880 nm.

**Figure 4:**
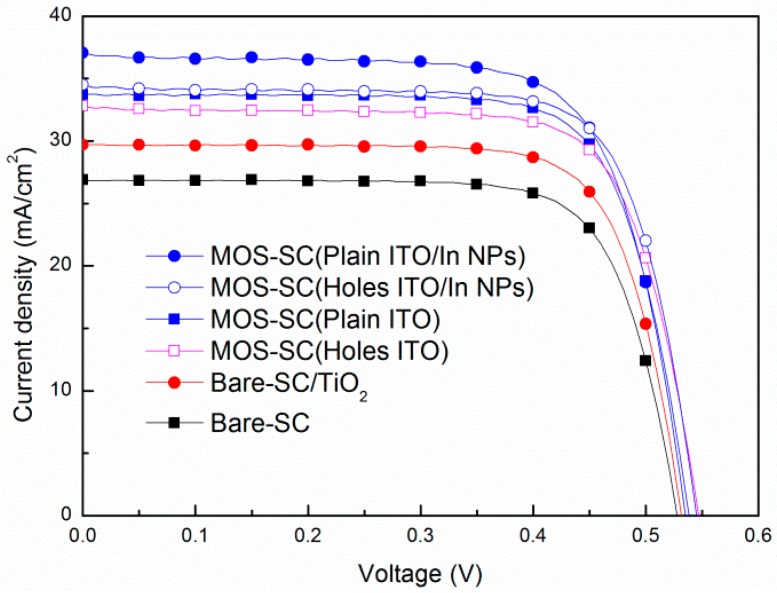
Photovoltaic J-V curves of all solar cells tested in this study (without applied bias voltage).

Figure 4 presents the photovoltaic J-V curves of the bare cell, the cell with a film of TiO_2_, the MOS cell with a plain ITO electrode, the MOS cell with a perforated ITO electrode, and the MOS cell with In-NPs and a plain ITO electrode, and the MOS cell with In-NPs and a perforated ITO electrode. Table 2 lists the statistics data of the photovoltaic performance of the solar cells evaluated in this study. The short-circuit current density (*J*_sc_) values of the cell with a layer of TiO_2_ (27.99, 29.65 mA/cm^2^) and the cell with an ITO/TiO_2_-layer structure (32.81, 33.71 mA/cm^2^) were higher than that of the bare cell (26.35, 26.88 mA/cm^2^), due to the anti-reflective properties of the TiO_2_ and ITO/TiO_2_. The *J*_sc_ of the MOS cell with a plain ITO electrode (33.71 mA/cm^2^, at 0 V bias) was higher than the cell with a perforated ITO electrode (32.81 mA/cm^2^, at 0 V bias) due to the large area of ITO and its anti-reflective properties. Clearly, the improvements in *J*_sc_ are proportional to the improvements in EQE as well as reduced reflectance. The improvement in *J*_sc_ provided by the In-NPs was 9.93% in the MOS cell with a plain ITO electrode and In-NPs (from 33.71 to 37.06 mA/cm^2^, under a bias voltage of 0 V), which is greater than the 5.18% of the cell with a perforated electrode (from 32.81 to 34.51 mA/cm^2^, under a bias voltage of 0 V).

**Figure 5:**
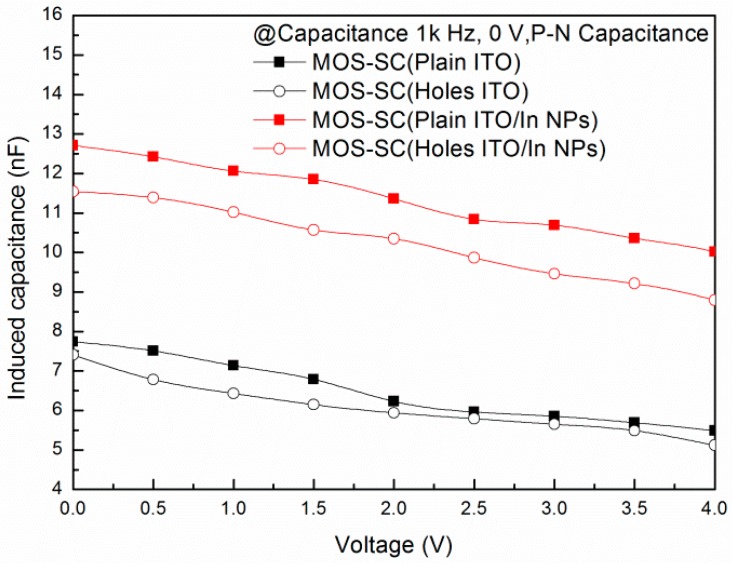
Induced capacitance of all MOS cells: measurements obtained at the front Al/Ti electrode and back Al electrode are a function of voltage applied to the ITO electrode.

Figure 5 presents the junction capacitance of all MOS cells. Measurements obtained at the front Al/Ti electrode and back Al electrode are a function of voltage applied to the ITO electrode. We observed a decrease in the induced capacitance with an increase in the bias voltage. These results indicate that the depletion width under the ITO region was enlarged. The induced capacitance of the MOS cell with a perforated ITO electrode was less than that of the cell with the plain ITO electrode due to a reduction in area. The large capacitance on all cells with In-NPs can be attributed to an increase in the surface area with integrated metallic particles.

**Figure 6:**
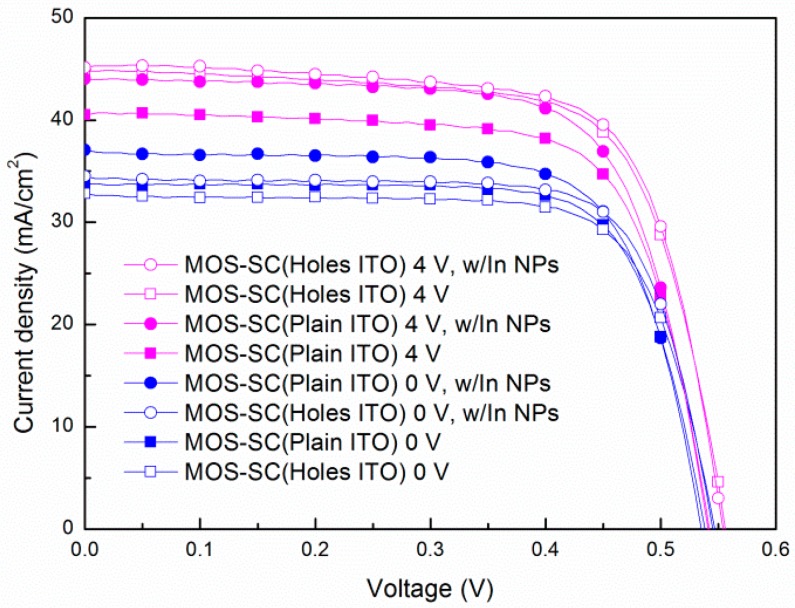
Photovoltaic J-V curves of MOS cells with and without In-NPs as a function of voltage applied to the ITO electrode (0 and 4 V).

Figure 6 presents photovoltaic J-V curves of the proposed MOS cell with both types of ITO electrodes (with and without In-NPs) and with and without bias voltage. The *J*_sc_ of the MOS cell with a perforated ITO electrode (44.84 mA/cm^2^, under a bias voltage of 4 V) was higher than the cell with a plain ITO electrode (40.49 mA/cm^2^, under a bias voltage of 4 V). The *J*_sc_ enhancements of 36.66% (from 32.81 to 44.84 mA/cm^2^) and 20.16% (from 33.71 to 40.49 mA/cm^2^) corresponding to the above-mentioned conditions were obtained due to the effects of the bias voltage. Furthermore, the *J*_sc_ of the MOS-structure cell with perforated ITO and In-NPs (45.13 mA/cm^2^, under a bias voltage of 4 V) was higher than that with the plain ITO and In-NPs (44.04 mA/cm^2^, under a bias voltage of 4 V) and the *J*_sc_ enhancements of 37.54% (from 32.81 to 45.13 mA/cm^2^) and 36.66% (from 33.71 to 44.04 mA/cm^2^) corresponding to the above-mentioned conditions can be attributed to plasmonic light scattering as well as biasing effects. It is interesting to note that under voltage bias, the improvements in *J*_sc_ are more pronounced in the MOS cell with a perforated ITO electrode than in the cell with a plain ITO electrode. Surface plasmon polaritons (SPPs) travel along ITO/TiO_2_ and the localized surface plasmon resonance (LSPR) of In-NPs can be attributed to efficient coupling and the scattering of incident light into the active layer of the cells. However, the underlying mechanism has yet to be fully elucidated and is undergoing further study.

## 3. Materials and Methods 

### 3.1. Bare-Type P-N Silicon Solar Cell

A 525-μm-thick, boron-doped C-Si wafer with resistivity of 10 Ω·cm and (100) orientation was used as a substrate in this study. The wafers were cut into samples of 10 × 10 mm^2^ for the fabrication of silicon solar cells. After RCA (Radio Corporation of America) cleaning, an n^+^-Si emitter layer with sheet resistance of approximately 80 Ω/sq was applied to the C-Si wafer using a phosphor diffusion process in a rapid thermal annealing (RTA) chamber (eRTP-MV, Giant Technology Corporation, Taiwan) at 900 °C for 2 min. The oxide layer on the surface of the wafer was removed using hydrogen fluoride (HF) solution. Following the phosphor diffusion of the n^+^-Si emitter layer, a 200 nm Al film was deposited on the rear surface using electron-beam (e-beam) evaporation (EVM-8, Kao Duen Technology Corporation, New Taipei City, Taiwan) and then annealed at 450 °C for 5 min to form the back electrode with good ohmic contact. Finally, top-contact grid-electrodes were formed using a 20 nm Ti film and 300 nm Al film using photolithography lift-off and e-beam evaporation to create bare-type p-n Si solar cells. These bare-type Si solar cells (denoted as bare cells) were used as a reference with regard to optical reflectance (Lambda 35, PerkinElmer Inc., Waltham, MA, USA), dark current-voltage (I-V, HP 4145B, Hewlett Packard, Palo Alto, CA, USA), external quantum efficiency (Enli Technology Co. Ltd., Kaohsiung, Taiwan), and photovoltaic I-V under air mass (AM) 1.5 G illumination. The solar simulator (XES-151S, San-Ei Electric Co. Ltd., Osaka, Japan) was calibrated using a National Renewable Energy Laboratory (NREL)-certified crystalline silicon reference cell (PVM-894, PV Measurements Inc., Boulder, CO, USA) prior to measurement.

### 3.2. MOS-Structure Silicon Solar Cell

E-beam evaporation was then used to deposit a 20-nm-thick TiO_2_ film on the surface of the bare solar cell. This was followed by a 50-nm-thick ITO film using thermal sputtering (PSUVH-450, HOPE Vacuum Technology Co. Ltd., Chai-yi, Taiwan) over spaced Al-finger electrodes to construct an ITO electrode. Lift-off photolithographic processing resulted in the formation of an ITO-TiO_2_-Si structure, denoted as the MOS-structure solar cell. We also fabricated samples using a plain-type ITO electrode as well as samples with ITO electrodes with periodic holes referred to as perforations (these were added using lift-off photo-resister patterns). The holes on the ITO electrode formed a matrix-configuration of 2 × 88, wherein the hole diameter was 10 μm and hole spacing was 39 μm. We then measured the dark I-V curves, optical reflectance, EQE, and photovoltaic current density-voltage (J-V) of the MOS-structure cell, to enable comparison with the bare solar cell. These measurements further elucidated the passivation and anti-reflection effects of the ITO/TiO_2_ layer-structure. We also compared cells with plain ITO electrodes and perforated ITO electrodes with regard to electric and optical performance. Enhancements in photovoltaic performance and reductions in p-n junction capacitance were indicated by photovoltaic J-V and C-V measurements (HP 4274A, Hewlett Packard), during which bias voltage was applied to the ITO electrode. 

### 3.3. MOS-Structure Silicon Solar Cell with In-NPs

**Figure 7:**
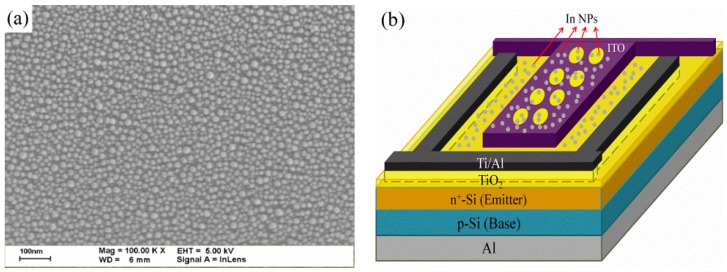
(**a**) SEM images showing the size and profile of In-NPs; (**b**) Schematic diagram of proposed MOS solar cell with In-NPs and perforated ITO-electrode.

Finally, we deposited a 3.8-nm-thick film of indium (In) on the surface of the MOS-structure solar cells using e-beam evaporation followed by annealing at 200 °C for 10 min in a rapid thermal annealing chamber under ambient H_2_ to produce In-NPs. Figure 7a presents a Scanning electron microscopy (SEM; Hitachi S-4700, Hitachi High-Tech Fielding Corporation, Tokyo, Japan) image of In-NPs profile deposited on ITO surface. The average diameter and coverage of the In-NPs were 17.70 nm and 30.5%, respectively. The objective was to evaluate the effect of plasmonic scattering induced by the In-NPs on the performance of MOS cells. We also sought to evaluate the performance of MOS cells with In-NPs with and without applied bias voltage based on photovoltaic J-V measurements. Figure 7b illustrates the proposed MOS-structure solar cell with In-NPs and a perforated ITO-electrode. 

## 4. Conclusions 

This paper reports MOS-structure silicon solar cells with plain ITO electrodes or perforated ITO electrodes (with or without plasmonic In-NPs) under an applied bias voltage of 0 to 4 V. The efficiency of the MOS-structure cells with perforated ITO electrodes (17.80%) is superior to that of cells with plain ITO electrodes (16.87%) and coated In-NPs under a bias voltage of 4 V. It appears that the application of bias voltage to the ITO electrode increased the depletion width and enhanced the collection efficiency of photo-carriers. Moreover, surface plasmon polaritons along the ITO/TiO_2_ as well as the localized surface plasmon resonance of In-NPs are responsible for the efficient coupling and scattering of incident light into the active layer of the cells, resulting in enhanced efficiency.

## Figures and Tables

**Table 1:** Ideality factor (*n*) and reverse saturation current density (*J*_0_) extracted from measured I-V curves at 25 °C.

Cell Type	*n*	*J*_0_ (A/cm^2^)
Bare SC	1.87	6.14 × 10^−8^
MOS SC (Plain ITO)	1.51	3.84 × 10^−9^
MOS SC (Holes ITO)	1.65	1.82 × 10^−8^

**Table 2:** Summary of photovoltaic performance of all solar cells evaluated in this study.

		Cell #	Bare SC	TiO_2_/SC	MOS SC at 0 V	MOS SC at 4 V	MOS SC w/In NPs, at 0 V	MOS SC w/In NPs, at 4 V
*V*_oc_ (mV) ^a^	ITO (Holes)	Cell-1	539.3	543.2	547.0	557.6	545.1	554.3
		Cell-2	533.8	541.1	543.2	553.2	542.6	552.4
		Average	536.6	542.2	545.1	555.4	543.9	553.4
	ITO (Plain)	Cell-1	527.9	531.8	538.4	542.7	535.3	541.2
		Cell-2	526.6	532.2	539.7	544.3	537.2	542.8
		Average	527.3	532.0	539.1	543.5	536.3	542.0
*J*_sc_ (mA/cm^2^)	ITO (Holes)	Cell-1	26.35	27.99	32.81	44.84	34.51	45.13
		Cell-2	26.82	28.53	33.07	45.98	34.84	46.38
		Average	26.59	28.26	32.94	45.41	34.68	45.76
	ITO (Plain)	Cell-1	26.88	29.69	33.71	40.49	37.06	44.04
		Cell-2	26.38	28.81	33.15	40.12	36.47	43.52
		Average	26.63	27.75	33.43	40.31	36.77	43.78
*FF* (%) ^b^	ITO (Holes)	Cell-1	75.4	75.8	73.8	70.1	74.5	71.1
		Cell-2	75.0	75.1	74.3	69.4	74.9	69.8
		Average	75.2	75.5	74.1	69.8	74.7	70.5
	ITO (Plain)	Cell-1	74.6	75.1	74.4	71.9	71.7	70.8
		Cell-2	75.1	75.4	74.8	72.0	72.3	70.4
		Average	74.9	75.3	74.6	72.0	72.0	70.6
η (%) ^c^	ITO (Holes)	Cell-1	10.72	11.53	13.24	17.53	14.01	17.80
		Cell-2	10.74	11.59	13.35	17.64	14.16	17.88
		Average	10.73	11.56	13.30	17.59	14.09	17.84
	ITO (Plain)	Cell-1	10.59	11.85	13.50	15.80	14.23	16.87
		Cell-2	10.43	11.56	13.38	15.72	14.16	16.63
		Average	10.51	11.71	13.44	15.76	14.20	16.75

^a^ *V*_oc_: open-circuit voltage; ^b^ *FF*: fill factor; ^c^ η: conversion efficiency.
